# Noninvasive Assessment of the Effect of Position and Exercise on Pulse Arrival to Peripheral Vascular Beds in Healthy Volunteers

**DOI:** 10.3389/fphys.2017.00047

**Published:** 2017-02-06

**Authors:** Yurie Obata, Qi J. Ong, J. T. Magruder, Helen Grichkevitch, Dan E. Berkowitz, Daniel Nyhan, Jochen Steppan, Viachaslau Barodka

**Affiliations:** ^1^Division of Cardiac Anesthesia, Department of Anesthesiology and Critical Care Medicine, Johns Hopkins University School of MedicineBaltimore, MD, USA; ^2^Newcastle University School of MedicineNewcastle, UK; ^3^Division of Cardiac Surgery, Department of Surgery, Johns Hopkins University School of MedicineBaltimore, MD, USA

**Keywords:** pulse arrival time, plethysmograph, body position, exercise, pulse wave velocity

## Abstract

**Background:** The effects of position and exercise on pulse wave distribution across a healthy, compliant arterial tree are not fully understood. We studied the effects of exercise and position on the pattern of pulse arrival times (PATs) in healthy volunteers. Moreover, we compared the pulse arrival time ratios to the respective distance ratios between different locations.

**Methods:** Thirteen young healthy volunteers were studied, using an electrocardiogram and plethysmograph to simultaneously record pulse wave arrival at the ear lobe, index finger and big toe. We compared the differences in PAT between each location at rest and post-exercise in the supine, sitting, and standing position. We also compared the PAT ratio (toe/ear, toe/finger, and finger/ear) to the corresponding pulse path distance ratios.

**Results:** PAT was shortest at the ear then finger and longest at the toe regardless of position or exercise status. PATs were shorter post-exercise compared to rest. When transitioning from a standing to sitting or supine position, PAT to the ear decreased, while the PAT to the toe increased, and PAT to the finger didn't significantly change. PAT ratios were significantly smaller than predicted by the path distance ratios regardless of position or exercise status.

**Conclusions:** Exercise makes PATs shorter. Standing position decrease PAT to the toe and increase to the ear. We conclude that PAT and PAT ratio represent the arterial vascular tree properties as surely as pulse transit time and pulse wave velocity.

## Introduction

When the heart contracts it ejects a bolus of blood, the stroke volume, into the arterial vascular system, which is then distributed to the peripheral tissues. The time period needed for a pulse wave to travel from the heart to the peripheral tissues depends on the distance traveled and the velocity of the pulse wave, which in turn is determined by various physical properties such as vessel diameter, wall thickness, and compliance (Bramwell and Hill, 1922).

Pulse wave velocity (PWV) has been studied extensively as a predictor for adverse cardiovascular events (Steppan et al., 2011). PWV is the ratio of vascular path length and the pulse transit time (PTT) between two sites of measurement. Pulse arrival to the peripheral vascular beds, the PTT, is the time from aortic valve opening till the foot of the pulse waveform at the peripheral site. Since it is impossible to directly measure aortic valve opening from an ECG alone, Pulse arrival time (PAT) has been widely used instead of PTT. However, these two are distinctly different. PAT is defined as the time delay between the peak of the R wave of the ECG waveform and the arrival (upstroke) of the arterial pulse wave in the periphery. Hence, PAT includes both pre-ejection time (ventricular electromechanical delay plus isovolumic contraction time) and PTT (Zhang et al., 2011).

Prior studies reported the PAT to different peripheral vascular beds in subjects of different age, height, and with different blood pressures (Allen and Murray, 2002; Nitzan et al., 2002). The PAT was consistently shortest at the ears, and longest at the toe. They showed that higher blood pressures and age were associated with shortening of the PAT, suggesting that PWV differs in distinctive peripheral vascular beds (Nitzan et al., 2002). Indeed Liu et al. showed that PWV is highest between the heart and toes, a slightly lower between the heart and fingers, and lowest between the heart and earlobe (Liu et al., 2011). In this manuscript, we studied the effects of position and exercise on the PAT to different parts of the body. Changes in body position triggers multiple cardiovascular responses due to the changes in hydrostatic pressure and sympathetic activity. In addition, exercise affects vascular properties through both vasodilation of arterioles and increased vasoconstriction due to sympathetic stimulation (Sharman et al., 2005; Roy and Secomb, 2014). Moreover, we compared the pulse arrival ratios to the respective distance ratios between different locations.

Specifically, we examined the PATs to three different vascular beds (ear lobe, index finger, and big toe) for the same heartbeat in three different positions (standing, sitting, and supine) at rest and post-exercise in young healthy subjects, as these factors might significantly affect PAT at different peripheral vascular beds.

## Materials and methods

This study was approved by The Johns Hopkins Medicine Institutional Review Board (IRB00074229). We enrolled 13 healthy volunteers with no history of vascular or cardiac disease, age 23–41 years old. Recruitment was done through e-mail or word of mouth in accordance with the Internal Review Board consent scripts. Inclusion criteria were: Healthy adults, age 21–50 years, both genders. Exclusion criteria were: Subject refusal to participate, known cardiovascular disease of any kind, pregnancy, and any disability preventing mild physical exertion. Two subjects who joined the study were excluded. The first subject was excluded due to the inability to finish the study protocol. The second subject was excluded as we were unable to obtain a plethysmograph signal on the toes. After verifying that each subject had no restrictions to participate in the study, we measured and recorded each subject's weight, half wingspan (distance from sternal notch to index finger with arm in 90° lateral extension), and self-reported height.

### Study protocol

A standard three lead electrocardiogram (ECG; Bio Amp FE132, ADInstruments, Australia) was placed for continuous monitoring of electrical cardiac activity. Next, we placed capillary plethysmograph sensors [MLT1020EC IR Plethysmograph (ear), MLT1020PPG IR Plethysmograph (finger and toe), ADInstruments, Australia] on both left and right sides for each of the following locations: ear lobes in a standing position, index fingers in a sitting position with hands hanging free by their sides, and on the big toes lying in the supine position. We simultaneously recorded the ECG and plethysmograph bilaterally for each location (ear, index finger, big toe). Then, we simultaneously recorded the ECG along with the plethysmographs from one unilateral ear, finger, and big toe for 30 s each in the standing, sitting, and supine position. The ECG and plethysmograph sensors were then removed from the subjects and a blood pressure cuff applied to record blood pressure in the standing, sitting and prone position respectively. For the exercise part of the experiment the subjects were required to perform 30 squats. The ECG and plethysmograph sensors were reattached to the subject's left ear lobe, index finger, big toe, and the recording redone in the standing, sitting, and supine positions. All data for the “post-exercise” portion was collected within 3 min of the subject completing 30 squats. The ECG and plethysmograph signals were simultaneously digitally converted at 1 kHz (PowerLab 4/26, ADInsruments, Australia) and analyzed by eye and hand (LabChart 8.0, ADInstruments, Australia). The PowerLab has intrinsic filters built in the Bio Amp FE132 (ADInstrumens, Australia; see https://www.adinstruments.com/products/PowerLab). Since all signals were recorded simultaneously and through the same AD converter (PowerLab 4/26, ADInstruments, Australia) there should be no time delays according to the manufacture.

### Data extraction

**Figure 1:**
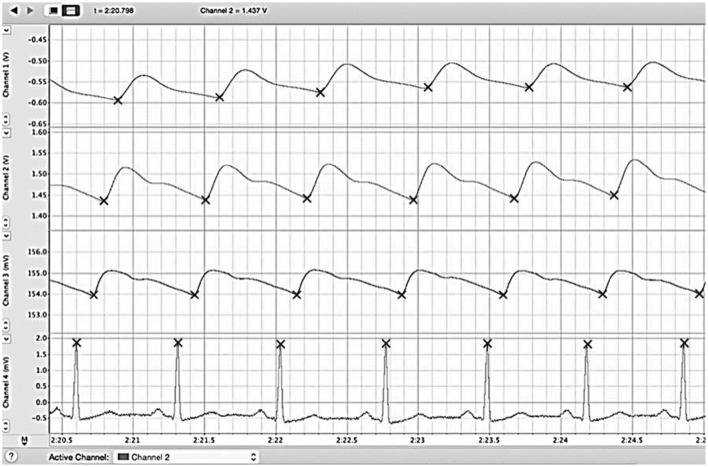
**Example reading of LabChart file**. We simultaneously recorded the ECG (bottom) along with plethysmographs from big toe (first from the top), finger (second from the top), and ear (third from the top) and for 30 s each in standing, sitting, and supine positions. The PAT to each location (ear lobe, index finger, and big toe) was assessed by calculating the time delay between the peak of the R wave on the ECG and the first subsequent positive inflection on the plethysmograph trace. Periods consisting of 10 consecutive heart beats from each position (standing, sitting, supine) before and after exercise were then used to calculated the mean PAT for the three locations (ear lobe, index finger, big toe). ECG, electrocardiogram; PAT, pulse arrival time.

From the data collected, the PAT to each location (ear lobe, index finger, and big toe) was assessed by calculating the time delay between the peak of the R wave on the ECG and the first subsequent positive inflection on the plethysmograph tracing. To compare PATs to different tissue beds from the same heartbeat, we took the corresponding R wave of the ECG, which was assigned a time value of zero. The representative data is shown in Figure 1. Periods consisting of 10 consecutive heart beats from each position (standing, sitting, supine) before and after exercise were then used to calculate the mean PAT for the three locations (ear lobe, index finger, big toe). To present the relative time differences between PAT to either ear, finger, or toe; we calculated the PAT ratios, toe/ear, toe/finger, and finger/ear. The collected data was then tabulated and used for statistical analysis.

### Statistical analyses

Data was analyzed using GraphPad Prism version 6.0 (GraphPad Software, San Diego, California, USA). Continuous variables are reported as mean ± standard deviation (*SD*). A paired *t*-test was used to compare the PATs on the left side with the PATs in the right side and to compare the PATs at rest with the PATs post-exercise at each location and in each position. Repeated-measures analysis of variance (ANOVA) with a Tukey test were used for multiple comparisons between PATs measured at different locations and positions as well as PAT ratios between different locations. Repeated-measures ANOVA with a Dunnett test were used for multiple comparisons between the path distance ratios and PAT ratios in different positions. The threshold for statistical significance was chosen to be *P* < 0.05.

## Results

Demographics and baseline characteristics of the volunteers are presented in Table 1. Average age was 30 years and ranged from 23 to 41 years. Females made up 45% of the cohort and males 55%. At rest systolic blood pressure was 114 ± 13 mmHg and diastolic blood pressure 66 ± 7 mmHg.

**Table 1:** **Cohort demographics and baseline characteristics**.

**Subject**	**Weight (kg)**	**Height (cm)**	**Distance Ratio**			**SBP (mmHg)**	**DBP (mmHg)**	**Age (years)**	**Gender**
			**Toe/Ear**	**Toe/Finger**	**Finger/Ear**				
1	80.5	188	5.9	1.8	3.4	113	64	23	M
2	57.5	163	5.6	1.7	3.4	108	68	36	F
3	63.0	174	6.0	1.9	3.2	110	63	26	F
4	95.5	177	5.8	1.8	3.3	142	84	39	M
5	54.2	164	5.5	1.7	3.2	111	67	36	F
6	79.5	175	5.7	1.7	3.4	116	62	31	M
7	82.1	174	5.7	1.7	3.3	113	64	41	M
8	47.7	165	5.5	1.7	3.3	98	61	23	F
9	60.1	171	5.6	1.7	3.3	95	58	23	F
10	69.5	173	5.7	1.7	3.3	117	69	29	M
11	94.6	178	5.8	1.7	3.4	128	68	26	M
Mean	71.3	173	5.7	1.7	3.3	114	66	30	
*SD*	16.2	7	0.2	0.1	0.1	13	7	7	

*DBP, diastolic blood pressure; SBP, systolic blood pressure; SD, standard deviation*.

**Figure 2:**
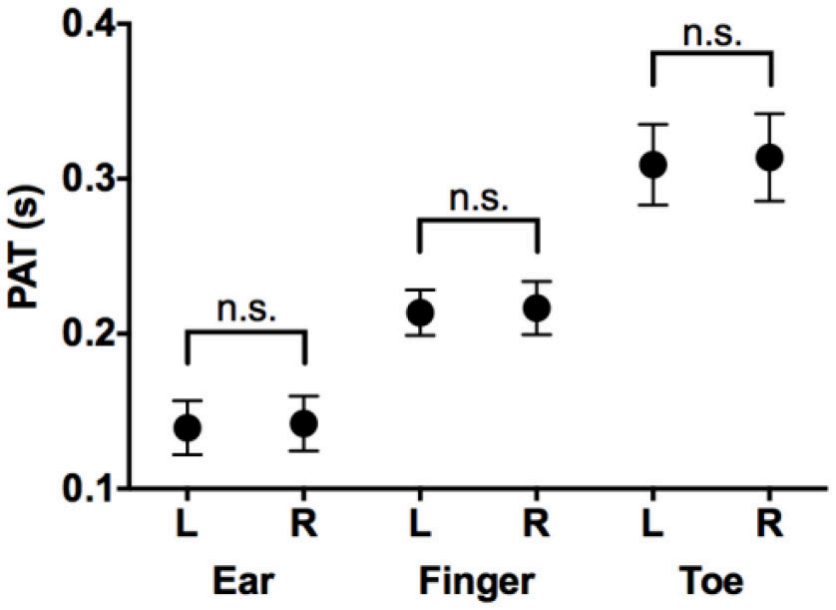
**Comparison of PATs measured in left side and those in right side**. Mean and *SD* in PAT at ear, finger, and toe in left and right sides. PAT, pulse arrival time; L, left side; R, right side; n.s., not significant.

PATs at each location (ears, toes, fingers) were not significantly different for the left and right side (*p* = 0.49 at ear, *p* = 0.22 at finger, *p* = 0.39 at toe; Figure 2). We therefore elected to compare only one side (left) to measure the PATs at different locations.

Mean and *SD* of the PATs and PAT ratios are tabulated in Table 2.

**Table 2:** **Effect of exercise on Cohort mean PATs and PAT ratios of all three positions**.

**PAT (s)**				**PAT ratio**			
**Standing**	**At rest**	**Post-exercise**	***P*-value**	**Standing**	**At rest**	**Post-exercise**	***P*-value**
Ear	0.15 ± 0.02	0.12 ± 0.01	<**0.001**	Toe/Ear	1.84 ± 0.20	2.10 ± 0.19	<**0.001**
Finger	0.21 ± 0.02	0.19 ± 0.03	0.154	Toe/Finger	1.28 ± 0.10	1.32 ± 0.13	0.325
Toe	0.26 ± 0.02	0.25 ± 0.02	**0.043**	Finger/Ear	1.43 ± 0.11	1.60 ± 0.23	**0.044**
**Sitting**	**At Rest**	**Post-exercise**	***P*****-value**	**Sitting**	**At rest**	**Post-exercise**	***P*****-value**
Ear	0.14 ± 0.02	0.12 ± 0.01	<**0.001**	Toe/Ear	1.98 ± 0.27	2.11 ± 0.20	0.089
Finger	0.20 ± 0.01	0.18 ± 0.01	<**0.001**	Toe/Finger	1.33 ± 0.11	1.38 ± 0.06	0.176
Toe	0.26 ± 0.02	0.25 ± 0.01	**0.016**	Finger/Ear	1.49 ± 0.19	1.53 ± 0.15	0.291
**Supine**	**At rest**	**Post-exercise**	***P*****-value**	**Supine**	**At rest**	**Post-exercise**	***P*****-value**
Ear	0.12 ± 0.02	0.11 ± 0.01	0.177	Toe/Ear	2.49 ± 0.32	2.55 ± 0.26	0.488
Finger	0.20 ± 0.01	0.18 ± 0.01	**0.003**	Toe/Finger	1.47 ± 0.10	1.57 ± 0.16	**0.026**
Toe	0.29 ± 0.02	0.29 ± 0.02	0.565	Finger/Ear	1.69 ± 0.21	1.63 ± 0.15	0.310

*PAT, pulse arrival time*.

*P-values are bold when P < 0.05*.

### Effects of location and exercise on pulse arrival (Figure 3)

In all positions, both at rest and post-exercise, the PATs between ear and toe were significantly different (shortest at the ear and longest at the toe).

**Figure 3:**
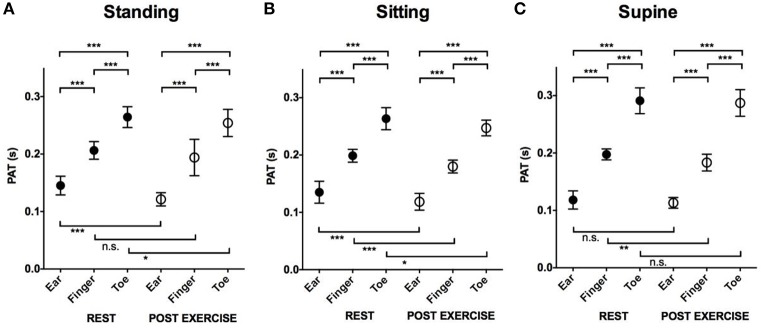
**The effect of location and exercise on the PAT**. Mean and *SD* in PAT at rest and post-exercise at different locations. **(A)** Comparison of PAT measured in the standing position. **(B)** Comparison of PAT measured in the sitting position. **(C)** Comparison of PAT measured in the supine position. ^***^P < 0.001; ^**^P < 0.01; ^*^P < 0.05; n.s., not significant. PAT, pulse arrival time; SD, standard deviation.

Post-exercise PATs at all three locations and in all three positions were shorter compared to PATs at rest although some of them were not statistically significant.

### Effects of position and exercise on pulse arrival at the ear (Figure 4A)

At rest, PAT to the ear was longest in the standing position (0.15 ± 0.02 s) compared to the sitting (0.14 ± 0.02 s, *p* = 0.03) and supine position (0.12 ± 0.02 s, *p* < 0.001). After exercise, PATs at the different locations were not significantly different (0.12 ± 0.01 s standing, 0.12 ± 0.01 s sitting, 0.11 ± 0.01 s supine).

**Figure 4:**
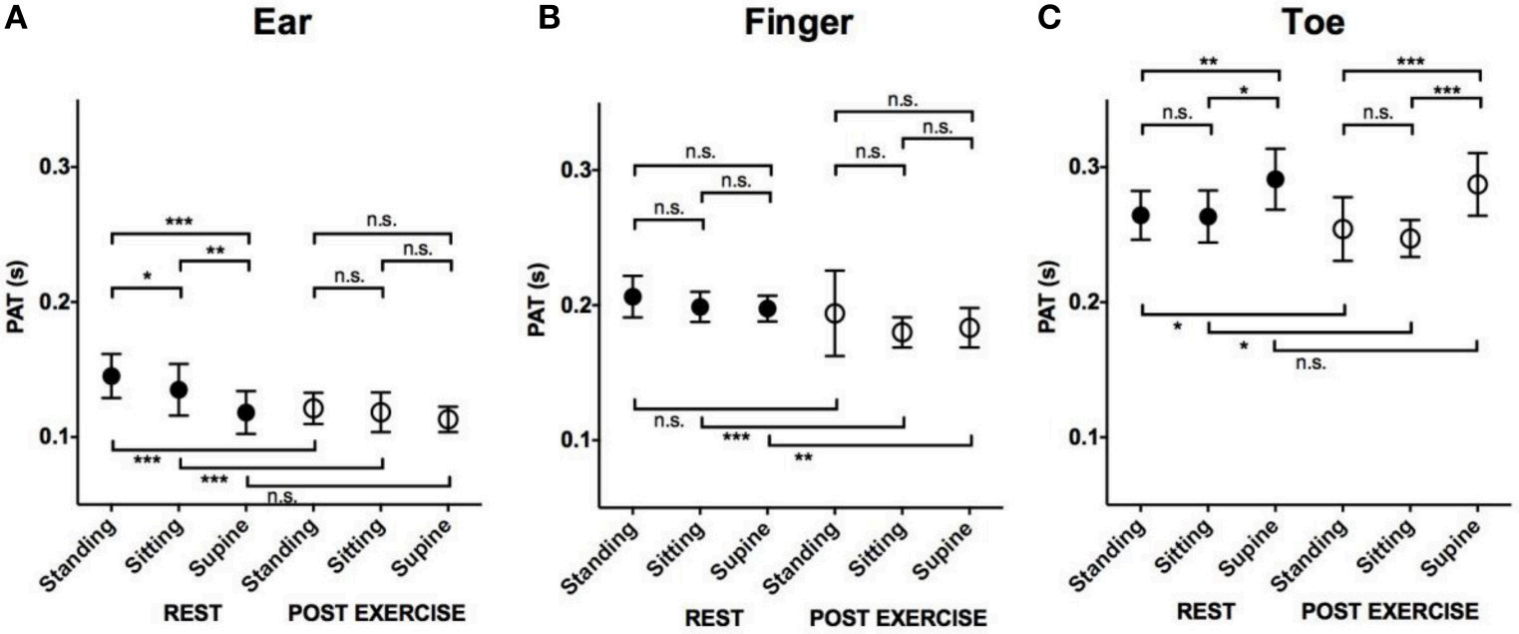
**The effect of position and exercise on the PAT**. Mean and *SD* in PAT at rest and post-exercise in different positions. **(A)** Comparison of PAT measured at ear. **(B)** Comparison of PAT measured at finger. **(C)** Comparison of PAT measured at Toe. ^***^P < 0.001; ^**^P < 0.01; ^*^P < 0.05; n.s., not significant. PAT, pulse arrival time; SD, standard deviation.

### Effects of position and exercise on pulse arrival at the finger (Figure 4B)

The effect of position on pulse arrival to the finger was minimal compared to the ear or toe. Both at rest and post-exercise, the PATs measured in different positions were not significantly different (at rest: 0.21 ± 0.02 s standing, 0.20 ± 0.01 s sitting, 0.20 ± 0.01 s supine, post-exercise: 0.19 ± 0.03 s standing, 0.18 ± 0.01 s sitting, 0.18 ± 0.01 s supine).

### Effects of position and exercise on pulse arrival at the toe (Figure 4C)

At rest, the PAT at the toe was longest in the supine position (0.29 ± 0.02 s) compared to standing (0.26 ± 0.02 s, *p* = 0.01) or sitting (0.26 ± 0.02 s, *p* = 0.03). The PATs in the standing and sitting positions were not significantly different (*p* = 0.98). Similarly, after exercise, the PAT was longest in the supine position (0.29 ± 0.02 s) compared to standing (0.25 ± 0.02 s, *p* < 0.001) or sitting (0.25 ± 0.01 s, *p* < 0.001; Figure 4C). The PATs in the standing and sitting positions were not significantly different (*p* = 0.46).

### Toe/ear PAT ratio and toe/ear pulse path distance ratio (Figure 5A)

Both at rest and post-exercise, the PAT ratio was significantly smaller than the pulse path distance ratio (5.7 ± 0.2), regardless of the position (*p* < 0.001).

**Figure 5:**
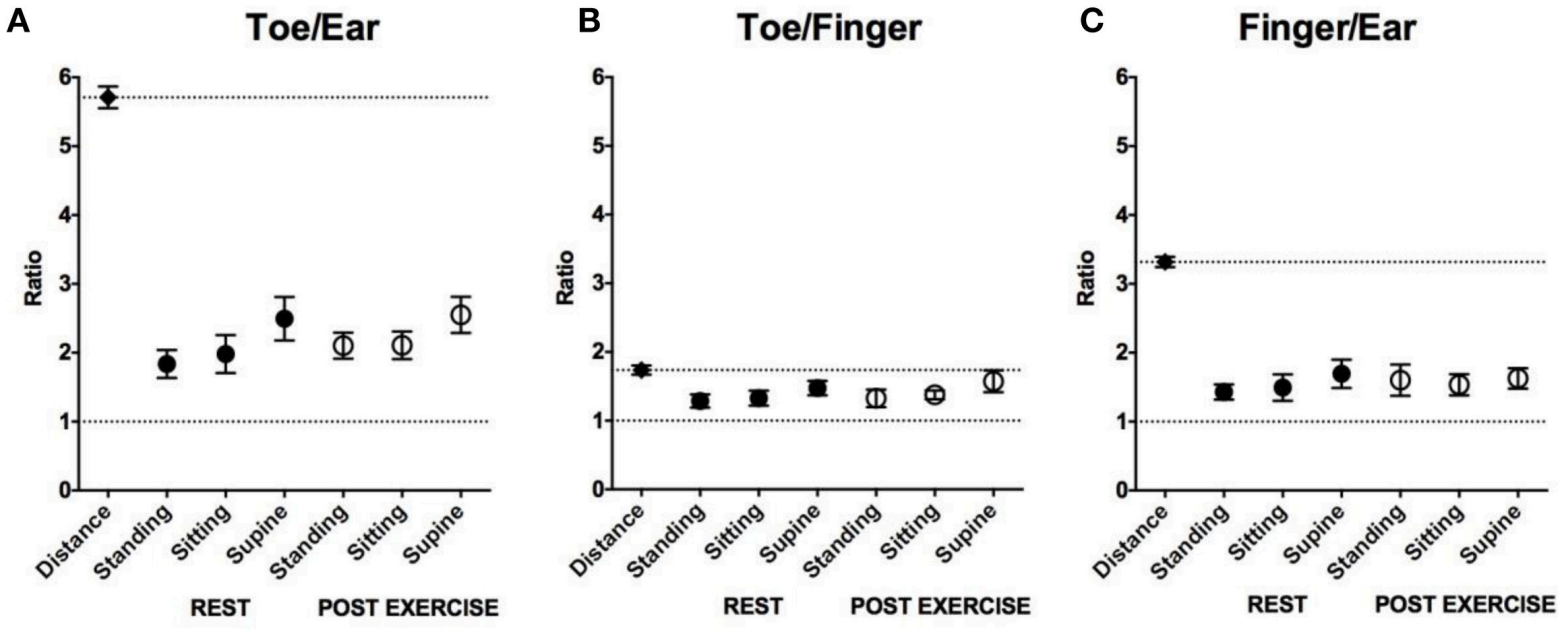
**Comparison of the path distance and the PAT ratio in the different positions. (A)** Comparison of mean and *SD* in toe/ear path distance ratio and those in toe/ear PAT ratio in the different positions. **(B)** Comparison of mean and *SD* in toe/finger path distance ratio and those in toe /finger PAT ratio in the different positions. **(C)** Comparison of mean and *SD* in finger/ear path distance ratio and those in finger/ear PAT ratio in the different positions. Across all positions PAT ratios were higher than 1 (a lower dashed line in each figure; *P* < 0.001) and shorter than the path distance ratio (an upper dashed line in each figure; *P* < 0.001–*P* < 0.05). PAT, pulse arrival time; SD, standard deviation.

At rest, the PAT ratio was smallest when standing (1.84 ± 0.20) compared to sitting (1.98 ± 0.27, *p* = 0.01) or supine (2.49 ± 0.32, *p* < 0.001).

After exercise, the PAT ratio was smaller when standing (2.10 ± 0.19) and sitting (2.11 ± 0.20) compared to being supine (2.55 ± 0.26, *p* < 0.001).

In the standing position, the PAT ratio was smaller at rest compared to post-exercise (*p* < 0.001). In the sitting and supine positions, the PAT ratios at rest were not significantly different compared to post-exercise (*p* = 0.09 and *p* = 0.49, respectively).

### Toe/finger PAT ratio and toe/finger pulse path distance ratio (Figure 5B)

Both at rest and post-exercise, the PAT ratio was significantly smaller than the pulse path distance ratio (1.7 ± 0.1), regardless of the position (*p* < 0.001).

At rest, the PAT ratio was smallest when standing (1.28 ± 0.10) compared to sitting (1.33 ± 0.11, *p* = 0.04) or supine (1.47 ± 0.32, *p* = 0.01).

After exercise, the PAT ratio was smaller when standing (1.32 ± 0.13) or sitting (1.38 ± 0.06) compared to supine (1.57 ± 0.16, *p* = 0.02: standing vs supine, *p* < 0.001: sitting vs. supine).

In both the standing and sitting position, the PAT ratios at rest were not significantly different compared to post-exercise (*p* = 0.33, *p* = 0.18, respectively). In the supine position, the PAT ratio was smaller at rest compared to post-exercise (*p* = 0.03).

### Finger/ear PAT ratio and finger/ear pulse path distance ratio (Figure 5C)

Both at rest and post-exercise, the PAT ratio was significantly smaller than the pulse path distance ratio (3.3 ± 0.1), regardless of the position (*p* < 0.001).

At rest, the PAT ratio was smaller when standing (1.43 ± 0.11) and sitting (1.49 ± 0.19) compared to being supine (1.69 ± 0.21, *p* < 0.001).

After exercise, the PAT ratios for standing (1.60 ± 0.23), sitting (1.53 ± 0.15), and being supine (1.63 ± 0.15) were not significantly different (*p* = 0.37).

In the standing position, the PAT ratio was smaller at rest compared to post-exercise (*p* = 0.04). In the sitting and supine positions, the PAT ratios at rest were not significantly different compared to post-exercise (*p* = 0.29 in sitting, *p* = 0.31 in supine).

## Discussion

The current study was performed in young healthy individuals to investigate the effects of position and exercise on the physiologic mechanisms of pulse wave distribution across a healthy, compliant arterial tree. Using noninvasive methods, we studied the pattern of pulse wave arrival at three distinct peripheral vascular beds at various positions and after exercise in healthy young volunteers.

Despite the fact that we used similar methodology as other groups, our study was focused on the effect of exercise and position on PAT, and we believe that our work is substantially different and adds new knowledge to the prior work published by Nitzan et al. (2002) and the group of Allen and Murray (2002). Moreover, to the best of our knowledge we are the first group that compared pulse arrival ratios to the respective distance ratios.

Similar to the studies mentioned above (Allen and Murray, 2002; Nitzan et al., 2002) we found that the pulse wave always arrives first at the ear, then at the index finger, and finally at the big toe—regardless of position or exercise status. This can be explained by the difference in the distances of the three locations from the heart. However, the PAT differences between distinct locations presented by the ratios of the PAT were relatively small compared to the distance ratios (Table 1). For example, the distance from the heart to the ear is on average 5.7 times shorter than to the toe, but the pulse arrives at the ear compared to the toe only 1.8–2.5 times sooner. This suggests that the arterial vascular tree actively maintains the smallest time difference in pulse wave arrival to the peripheral tissue beds irrespective of its distance from the heart. Moreover, the ratios are smaller for standing and sitting compared to the supine position, meaning that the PATs are more aligned in the vertical position compared to horizontal. We could speculate that a more simultaneous pulse arrival throughout the body is more important in the upright positions when people are likely to be more active, but we do not have any data to substantiate this claim.

Blood is transported along the arterial tree from the heart to peripheral tissues in order to supply those tissues' metabolic demand and to maintain homeostasis. This blood flow from the heart through the arterial system is provided in a pulsatile manner up to the pre-sphincter arterioles (Peterson, 1954). This allows the detection of a pulsatile waveform at the peripheral tissues, using plethysmography or pulse oximetry. Moreover, the time taken for the pulse wave to reach the vasculature of various peripheral tissue beds depends on a specific pulse propagation speed or pulse wave velocity (PWV) in each portion of the vascular tree (Bramwell and Hill, 1922). As one might infer, the distance of a particular vascular pathway always remains relatively constant, however PWV might undergo significant changes in particular arterial segments due to factors such as vascular tone and distending pressure (Bramwell and Hill, 1922). Since the arterial system does not contain valves, changes in position such as from lying to standing could hypothetically cause blood to pool in the lower extremities due to the formation of a hydrostatic gradient from the head to toe. In reality, in healthy individuals, the body produces a myogenic response, reacting to this change in hydrostatic pressure, by increasing the vascular tone through vasoconstriction in the lower extremities, leading to an increased distending pressure and wall tension in those arteries (Schubert and Mulvany, 1999). This increase in wall tension contributes toward an increased PWV and hence a shorter PAT (Bramwell and Hill, 1922; Learoyd and Taylor, 1966).

### Exercise

We hypothesized that the PAT shortens with exercise due to an increase in force of myocardial contraction, increased cardiac output, higher vascular wall tension, and central arterial wall stiffness (Strandell and Shepherd, 1967). Indeed our findings demonstrate that the PAT across all locations was shorter post-exercise. However, the pulse wave arrived relatively late at the lower extremity compared to the finger and ear as evidenced by increasing ratio of the toe/ear PAT and toe/finger PAT after exercise as compared to rest. One potential explanation is vasodilation in the metabolically active lower limbs and vasoconstriction in the less metabolically active upper limbs and head, to optimize metabolic/perfusion matching (Calbet and Joyner, 2010). Given that the subjects performed (squats) and therefore mainly utilized the muscles in the lower body, metabolic demand in the lower limb muscles would be higher than both the head and upper limbs. The net effect from the vasodilation in the lower limbs and vasoconstriction in the head and upper limbs was an increase in the toe/ear PAT ratios post-exercise. This is consistent with the fact that an increase in vascular tone (vasoconstriction) would increase PWV causing a decrease in PAT and vice versa (Bramwell and Hill, 1922; Sharman et al., 2005).

### Position

Our observations showed that a change in position from horizontal to vertical (supine to sitting or standing) led to a decrease in the PAT at the toe and an increase in the PAT at the ear. This might be explained by an increase in hydrostatic pressure in the arteries of the lower extremities and a decrease in the hydrostatic pressure in the arteries of the head and neck caused by the positional change from horizontal to vertical (Hasegawa and Rodbard, 1979). In healthy individuals, the vasculature adapts to the increase in hydrostatic pressure in the lower limbs by increasing wall tension or stiffness (Eiken et al., 2014). Without this response, blood would pool in the lower limbs and the perfusion of the upper limbs and head would be reduced. In many patients with orthostatic hypotension, disorders of the autonomic nervous system prevent this response from occurring, leading to reduced perfusion of the cerebral cortex and syncope (Stewart, 2002). Studies have shown that an increase in hydrostatic pressure and hence increased vascular wall tension causes an increase in PWV (Hasegawa and Rodbard, 1979). This explains our findings, wherein the PAT to the toes were shorter most likely due to the increase in wall tension and hence PWV, and the PAT to the ears were longer due to a decrease in wall tension and PWV when changing from a supine to standing position. However, overall, the PAT changes from lying supine to standing were not large. This might be due to fast adaptation by the vascular tone, autonomic nervous system signals, and myogenic responses as suggested previously (Pohl et al., 2000; Segal and Jacobs, 2001; Secomb, 2008; Roy and Secomb, 2014).

In our study, the pulse wave reached the ear before the index finger or big toe. This may be indicative of the body preferentially diverting blood to the cephalic region, although much more exploratory research is needed before coming to this conclusion. It is also unknown whether this pattern of pulse arrival is still true in individuals not included in the demographic studied (non-healthy individuals, children, or the elderly). The data collected in this study could be used as a baseline to which future studies can be compared against.

Our results are consistent with published findings of others. Liu et al. used ECG and photoplethysmography to measure PWV between different sites of the body in supine position (Liu et al., 2011). They found that PWV is highest between the heart and toes, a little lower between the heart and fingers and lowest between the heart and earlobe. They also report that PWV of the left and right sides were the same, which implies that pulse wave arrival time is identical to each location between the left and right. Nitzan et al. at all found that pulse arrival time to the toe is longer compared to the finger in supine position, which also supports our observation (Nitzan et al., 2002). Work of Allen and Murray on healthy subjects of different age, height, and with different blood pressures showed that PATs become shorter with increasing age and systolic blood pressure (Allen and Murray, 2002). Similar to our results they reported that PAT is shorter at the ear compared to the finger and toe in supine position. Our work expanded prior knowledge by focusing on the effects of exercise and position on PATs. Moreover, we compared the PAT ratios to their respective distance ratios. Our results suggest that the arterial vascular tree properties minimize the difference in the PAT between different peripheral tissues despite large changes in path distance.

### Limitations

The study was conducted on young and healthy volunteers and is therefore limited to this population. Our method of recruitment undoubtedly introduced a degree of sampling bias. The PowerLabs converter used was unable to distinguish between pressure generated by capillary filling and extremity movements; subjects had to stay very still while measurements were taken to ensure a readable trace. One subject who met all the inclusion criteria had to be excluded from the study because the equipment was unable to provide a readable toe plethysmograph trace. The PowerLabs hardware allowed us to record only four tracings at a time, such that we could not simultaneously record signals from both sides of the body. Rather, we had to do bilateral measurements first to confirm that each pulse arrived simultaneously to both sides at each level (toe, finger, and ear). For the exercise portion, we found it difficult to standardize the amount of physical activity based on our subjects various fitness and strength levels. Therefore, we felt squats would be an exercise that would be best suited to each individual's body weight and strength level. In the analysis of the data, we did not normalize for subjects' blood pressure or heart rate, which have been known to confound PWV (Lantelme et al., 2002). The choice of the peak of the R-wave on the ECG as the starting point of our PAT measurements also has its limitations as it includes the pre-ejection systolic phase of the ventricular contraction, the period between of isovolumetric ventricular contraction before the opening of the aortic valves (Geddes et al., 1981). However, the pre-ejection phase in healthy subjects is about 35 ms, whereas the PAT are in range of 150–300 ms, thus the potential error is in range of 15% (Biering-Sørensen et al., 2016). In future studies, the usage of a cardiac microphone to record the S1 heart sound could be more appropriate in obtaining a PAT sample.

## Conclusions

The main conclusions from this study are:

1. The pulse wave always arrives at the ear first, then at the index finger, and finally the big toe—regardless of position or exercise status. This can be explained by the difference in the distances of the three locations from the heart.
2. PATs are shorter post-exercise irrespective of the subject's position, most likely due to an exercise-induced increase in sympathetic activity leading to a global increase in PWV.
3. When transitioning from a supine to a sitting or standing position, the PAT to the ear increased, while the PAT to the toe decreased. This could be explained by an increase in vascular tone in the lower limbs in response to the increase in hydrostatic pressure during positional change from horizontal to vertical.
4. The PAT ratios between two locations (i.e., toe/ear and finger/ear) were less than half of their respective distance difference ratios. We conclude that the arterial vascular tree properties minimize the difference in the PAT between different peripheral tissues despite large changes in path distance.

Hence, we conclude that PAT and PAT ratio represent the arterial vascular tree properties as surely as pulse transit time and pulse wave velocity.

In the future, we anticipate studying PATs in aging and diseased populations, as well as with differential exercise regimens. It is known that both aging and cardiovascular disease significantly affect PWV, and the presenting mechanisms might be distorted in those groups. The current study could serve as the natural comparison group for future investigations in the elderly and in patients with cardiovascular disease.

## Author contributions

Study design: VB. Data acquisition: QO, HG, VB. Data analysis: QO, YO, JM, VB. Interpretation of data, drafting the work, and revising it: QO, YO, DB, DN, JS, VB.

### Conflict of interest statement

The authors declare that the research was conducted in the absence of any commercial or financial relationships that could be construed as a potential conflict of interest.

## Abbreviations

PAT: pulse arrival time
PWV: pulse wave velocity.
